# Synthesis and Polymerization Kinetics of Rigid Tricyanate Ester

**DOI:** 10.3390/polym13111686

**Published:** 2021-05-21

**Authors:** Andrey Galukhin, Roman Nosov, Ilya Nikolaev, Elena Melnikova, Daut Islamov, Sergey Vyazovkin

**Affiliations:** 1 Alexander Butlerov Institute of Chemistry, Kazan Federal University, Kremlevskaya Str. 18, 420008 Kazan, Russia; romanosow@mail.ru (R.N.); ilkamoe1995@yandex.ru (I.N.); mei270700@mail.ru (E.M.); 2 Arbuzov Institute of Organic and Physical Chemistry, FRC Kazan Scientific Center, Russian Academy of Sciences, 8 Arbuzov Street, 420088 Kazan, Russia; daut1989@mail.ru; 3 Department of Chemistry, University of Alabama at Birmingham, 901 S. 14th Street, Birmingham, AL 35294, USA

**Keywords:** cyanate esters, polymerization kinetics, vitrification, diffusion control, thermal analysis, isoconversional kinetic analysis

## Abstract

A new rigid tricyanate ester consisting of seven conjugated aromatic units is synthesized, and its structure is confirmed by X-ray analysis. This ester undergoes thermally stimulated polymerization in a liquid state. Conventional and temperature-modulated differential scanning calorimetry techniques are employed to study the polymerization kinetics. A transition of polymerization from a kinetic- to a diffusion-controlled regime is detected. Kinetic analysis is performed by combining isoconversional and model-based computations. It demonstrates that polymerization in the kinetically controlled regime of the present monomer can be described as a quasi-single-step, auto-catalytic, process. The diffusion contribution is parameterized by the Fournier model. Kinetic analysis is complemented by characterization of thermal properties of the corresponding polymerization product by means of thermogravimetric and thermomechanical analyses. Overall, the obtained experimental results are consistent with our hypothesis about the relation between the rigidity and functionality of the cyanate ester monomer, on the one hand, and its reactivity and glass transition temperature of the corresponding polymer, on the other hand.

## 1. Introduction

**Figure 1:**
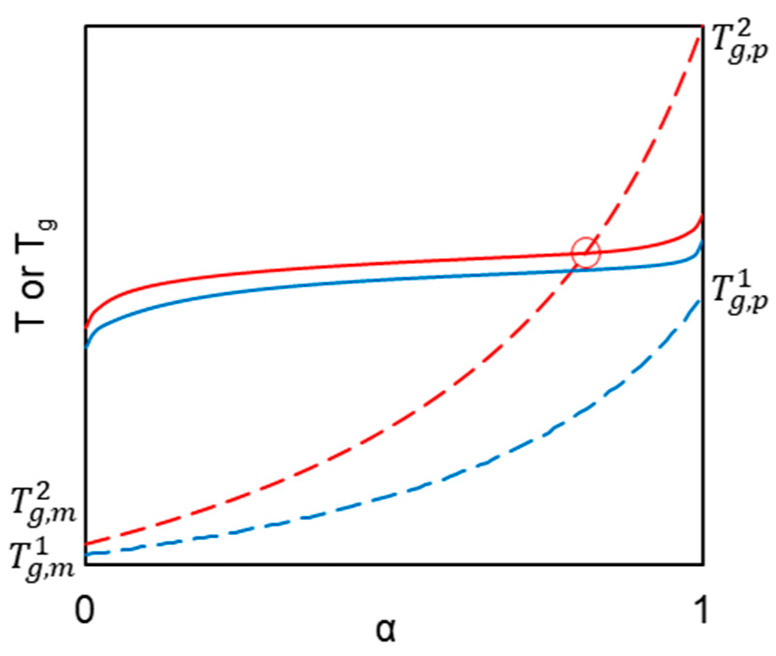
Schematic illustration of the effect of the polymer *T_g_* value on the reactivity of a monomer. Dashed lines represent variation of *T_g_* with conversion (*α*); solid lines represent *α–T* plot obtained at the heating rate β.

Unique mechanical, thermal, and electric properties of cyanate resins make them irreplaceable for electronic, military, and aerospace industries [1,2,3,4]. Maintaining the progress in creating high-performance cyanate resins with desired properties requires an understanding of fundamental relationships between the structure of the initial monomer, its reactivity, and properties of the final polymeric materials [3,5,6,7]. Finding such relationships is a non-trivial task because of the complex nature of the cyanate ester’s polymerization process [5,8]. For example, the well-known (in physical organic chemistry) Hammet-type correlations (i.e., the Hammet or Hammet–Taft equations), which describe electronic and steric effects of substitutions on the reactivity of compounds, [9] are not valid for multi-step processes because the rate constants of the individual reactions usually respond differently to substitutions [10]. For this reason, the application of such correlations is problematic for description of the cyanate esters reactivity. In addition, the reactivity of monomers in the condensed states (i.e., melt or solid) is affected by intermolecular interactions [11], which are not taken into account by the aforementioned equations. Furthermore, the kinetics of polymerization can be convoluted by a transition from a kinetic- to diffusion-controlled regime at later stages of the process [12], when the translational motion of the polymer chains slows down due to increasing viscosity of the reaction mixture. This transition is routinely linked to vitrification of the forming polymer. Therefore, one can expect that the structural factors that slow down the segmental mobility (i.e., those that increase the glass transition temperature, *T_g_*) should promote an earlier transition of the reaction kinetics to the diffusion-controlled regime during polymerization. This idea is illustrated schematically in Figure 1, where two monomers possess similar reactivity (similar *α–T* curves) but markedly different values for *T_g_*. It is seen that polymerization of a monomer that yields a polymer of larger *T_g_* is accompanied by vitrification (intersection of the corresponding *α–T* and *T_g_*–*α* curves), whereas polymerization that yields a polymer of lower *T_g_* does not cause vitrification. The glass transition temperature of cross-linked polymers depends on the cross-linking density [13,14], rigidity of the polymer network [15], and strength of intermolecular interactions [16]. Of these factors, the cross-linking density has the most straightforward effect that can be controlled in cyanate esters via the number of cyanate groups. Therefore, we can assume that rigid cyanate esters with three (or more) cyanate groups should yield polymers with larger *T_g_* values and, thus, should be more prone to transitioning to diffusion control during polymerization than dicyanate esters, especially the non-rigid ones.

Overall, our hypothesis is that polymerization of rigid n-functional cyanate esters with *n* > 2 should be affected by vitrification as described above and result in a polymeric product with a higher glass transition temperature than that of the cyanate esters of lower functionality and higher molecular flexibility. This hypothesis is tested by synthesizing a tricyanate ester based on a rigid triangular organic skeleton, which consists of seven conjugated aromatic units, and subjecting it to thermal polymerization. The monomer reactivity is probed by both conventional and temperature-modulated differential scanning calorimetry (DSC). The observed polymerization kinetics are treated in the frameworks of the isoconversional methodology [12]. The glass transition temperature and thermal stability of the polymerization product are tested by means of thermomechanical analysis and thermogravimetry, respectively, and compared with those of polymers obtained from commercially available cyanate esters based on bis-phenols.

## 2. Materials and Methods

**Figure 2:**
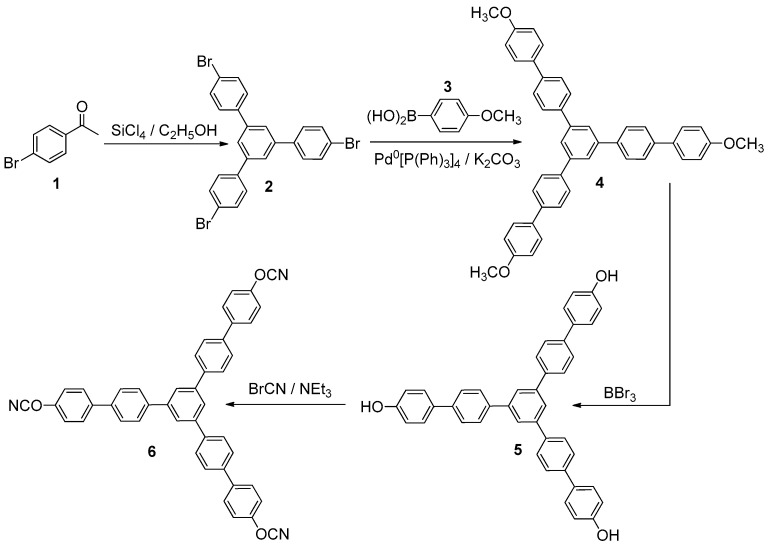
Schematic representation of the target cyanate ester **6** synthesis.

Dichloromethane (>99%, EKOS-1, Moscow, Russia), toluene (99.5%, EKOS-1, Moscow, Russia), acetone (>98%, Chimmed, Moscow, Russia), SiCl_4_ (99%, Sigma Aldrich, Saint Louis, MO, USA), tetrakis(triphenylphosphine)-palladium(0) (99%, Sigma Aldrich, Saint Louis, MO, USA), K_2_CO_3_ (>98%, EKOS-1, Moscow, Russia), n-butyllithium (2.5 M solution in hexane, Acros Organics, Waltham, MA, USA), triisopropyl borate (98+%, Acros Organics, Waltham, MA, USA), 4-bromanisole (>99%, Sigma Aldrich, Saint Louis, MO, USA), boron tribromide (99+%, Acros Organics, Waltham, MA, USA), triethylamine (>99%, Fisher Chemical, Moscow, Russia), cyanogen bromide (97%, Acros Organics, Waltham, MA, USA), Na_2_SO_4_ (anhydrous, >99.5%, Chimmed, Moscow, Russia), K_2_CO_3_ (98%, Chimmed, Moscow, Russia), 4-bromacetophenone (98+%, Acros Organics, Waltham, MA, USA), and SiO_2_ (60 Å, Machery-Nagel, Duren, Germany) were purchased and used without additional purification. High-performance liquid chromatography (HPLC) analysis indicated that the synthesized monomer was more than 99% pure. The 96% ethanol was distilled consecutively over CaO and CaH_2_ to produce absolute ethanol. Arium mini instrument (Sartorius, Goettingen, Germany) was used to generate deionized water (18.2 MΩ). Figure 2 schematically presents how the target cyanate ester was synthesized. The above synthetic approach follows the well-documented strategy [17,18,19,20] for obtaining cyanate esters, including those produced industrially.

Compounds **2–5** were synthesized according to known synthetic protocols described in [17,18,19].

1,3,5-*tris*-[4-(4-cyanatophenyl)phenyl]benzene (**6**). Cyanogene bromide (0.84 g, 7.85 mmol) and 1,3,5-tris [4-(4-hydroxyphenyl)phenyl]benzene (**5**) (0.51 g, 0.876 mmol) were mixed in anhydrous acetone and stirred at –30 °C; triethylamine (1.09 mL, 7.88 mmol) was dissolved in acetone and added dropwise to the aforementioned cooled mixture. Then, the reaction mixture was stirred at room temperature for 20 min, and white precipitate of triethylammonium salt was filtered off. Filtrate of the reaction mixture was evaporated. Dichloromethane was added to the residue, which was then washed with deionized water and dried over anhydrous sodium sulfate (Na_2_SO_4_). Silica gel column chromatography (dichloromethane eluent) was employed to purify the crude product. After removing the solvent, white crystals were obtained. Yield 71%. Melting point (DSC, 10 K/min): 273 °C. IR (cm^−1^): 2234, 2264 (-OCN functional group). ^1^H NMR (CDCl_3_): δ (ppm) 7.26–7.60 (12H, m, AA’BB’ spin system), 7.54–7.69 (12H, m, AA’BB’ spin system), 7.74 (3 H, s). ^13^C NMR (CDCl_3_): δ (ppm) 108.75 (C7), 152.36, 141.85, 140.53, 139.59, 138.63, 129.02, 127.97, 127.66, 125.23, 121.07, 115.78. Crystal Data for C_46_H_29_Cl_2_N_3_O_3_ (*M* = 742.62 g/mol): triclinic, space group P-1 (no. 2), *a* = 10.3354(2) Å, *b* = 11.4326(2) Å, *c* = 15.9093(2) Å, *α* = 107.324(2)°, *β* = 99.5630(10)°, *γ* = 94.157(2)°, *V* = 1754.72(5) Å^3^, *Z* = 2, *T* = 99.8(8) K, μ(Cu Kα) = 2.059 mm^−1^, *Dcalc* = 1.406 g/cm^3^, 21,578 reflections measured (5.936° ≤ 2Θ ≤ 153.09°), and 7081 unique (*R*_int_ = 0.0389, *R*_sigma_ = 0.0396), which were used in all calculations. The final *R*_1_ was 0.0650 (I > 2σ(I)), and *wR*_2_ was 0.2107 (all data). The CCDC number was 2077466.

**Methods for determination of structure and purity of target monomer.** A Rigaku XtaLab Synergy S instrument with a HyPix detector and a PhotonJet microfocus X-ray tube using Cu K_α_ (1.54184 Å) radiation was utilized to collect single crystal X-ray data for the monomer at low temperature. The CrysAlisPro data reduction package was used to index and integrate the images. Systematic errors and absorption were corrected using the ABSPACK module. The space group determination was accomplished with the GRAL module. The structure was solved with the aid of SHELXT. A refinement was carried out by a full-matrix least-squares analysis on F^2^ using SHELXL [21,22]. Non-hydrogen atoms were anisotropically refined. The hydrogen atoms were placed in the calculated positions and refined as riding atoms. The Mercury 4.1 program was used to generate the figures [23]. The target cyanate ester was crystallized from a dichloromethane–hexane mixture. A Dionex Ultimate 3000 chromatograph equipped with a UV detector (254 nm) and Dionex Acclaim 120 chromatographic column (C18-bonded silica, 5 µm, 120 Å, 4.6 mm × 250 mm) was employed to conduct HPLC analysis. The eluent was an acetonitrile–deionized water mixture (85 to 15 vol.%) that was introduced at a flow rate of 1 mL min^−1^. A Bruker AVANCE III NMR spectrometer operating at 600.13 MHz was used for the ^1^H and ^13^C analyses. A Bruker Vertex 70 FTIR spectrometer was utilized to record IR spectra.

Thermal analysis. A heat flux DSC 3+ (Mettler-Toledo) was employed to run calorimetric measurements. Temperature, heat flow, and tau-lag calibrations were conducted with the aid of in and Zn standards. The runs were performed under an 80 mL min^−1^ flow of Ar. The samples were placed into 40 µL aluminum pans closed with pierced lids and heated at the rates 5, 10, 15, and 20 °C min^−1^. Prior to the DSC measurements, dichloromethane was removed from the monomer crystals by heating in argon flow for 8 h at 80 °C, i.e., at ~40 °C above the boiling temperature of dichloromethane. The mass of the cyanate ester sample used was ~1 mg. The mass of the samples upon completion of the measurements decreased by a little over 1%. Temperature-modulated DSC (TMDSC) measurements were conducted by heating at 1 °C min^−1^ from ambient temperature to 400 °C. The linear temperature ramp was overlaid with stochastic temperature oscillations, with periods ranging from 15 to 30 s and the amplitude maintained at 0.5 °C. A Netzsch STA 449 F1 Jupiter thermal analyzer was employed for thermogravimetric analysis (TGA). The analysis was carried out by a heating of ~10 mg sample from 40 to 1000 °C under 75 mL min^−1^ argon flow. Thermomechanical analysis (TMA) was performed on a TMA 403 F1 Hyperion (Netzsch, Selb, Germany) dilatometer in a penetration mode. The sample was heated at 5 °C min^−1^ from 25 to 550 °C under nitrogen flow with an applied force of 2 N.

## 3. Computations

Kinetic analysis was performed in accordance with the recommendations of the ICTAC Kinetic Committee [24]. The dependence of the effective activation energy, *E_α_*, on conversion was determined by means of the flexible integral isoconversional method of Vyazovkin. The extents of conversion, *α*, were determined as the partial areas of the DSC peaks associated with polymerization of the cyanate ester. The Vyazovkin method eliminates a systematic error in E_α_ that arises when *E_α_* varies significantly with *α* [25]. This error is eliminated thanks to the flexible integration that presumes the constancy of *E_α_* only within a very narrow integration range, Δ*α* = 0.01. For each Δ*α*, *E_α_* is found by minimizing the following function:

(1) $$\Psi \left(E_{\alpha }\right)=\overset{p}{\underset{i=1}{\sum }}\overset{p}{\underset{j\neq i}{\sum }}\frac{J\left[E_{\alpha },T_{i}\left(t_{\alpha }\right)\right]}{J\left[E_{\alpha },T_{j}\left(t_{\alpha }\right)\right]}\;\;\;\;$$

where

(2) $$J\left[E_{\alpha },T_{i}\left(t_{\alpha }\right)\right]\equiv \overset{t_{\alpha }}{\underset{t_{\alpha -\Delta \alpha }}{\int }}\exp\left[\frac{-E_{\alpha }}{RT_{i}\left(t\right)}\right]dt\;\;\;\;$$

and *p* is the number of the temperature programs, *T*(*t*). The trapezoid rule was used to evaluate the integral. A minimum of Equation (1) was found by the COBYLA non-gradient method from the NLopt library. The uncertainties in the *E_α_* values were determined by means of a statistical procedure explained elsewhere [26].

A dependence of the pre-exponential factor on conversion was estimated by substituting the values of *E_α_* into the equation for the compensation effect:

(3) $$\ln A_{\alpha }=a+bE_{\alpha }\;\;\;\;$$

First, the *a* and *b* values were found by fitting the pairs of *lnA_i_* and *E_i_* into Equation (3). The *lnA_i_* and *E_i_* pairs were determined by substituting different reaction models, *f_i_*(*α*), into the linear form of the basic rate equation:

(4) $$\ln\left(\frac{d\alpha }{dt}\right)-\ln\left[f_{i}\left(\alpha \right)\right]=\ln A_{i}-\frac{E_{i}}{RT}\;\;\;$$

Substitution of each f_i_(α) model into Equation (4) yielded a corresponding pair of *lnA_i_* and *E_i_* values. Overall, five pairs of *lnA_i_* and *E_i_* were determined by using the model:

(5) $$f\left(\alpha \right)=\alpha^{m}{\left(1-\alpha \right)}^{n}\;\;$$

With five different combinations of *m* and *n* (*m* = 1, *n* = 1; *m* = 0.5, *n* = 1; *m* = 1, *n* = 0.5; *m* = 2, *n* = 1; *m* = 1, *n* = 2). This model was chosen because it imitates the autocatalytic reaction kinetics typically observed for cyanate esters polymerization [3]. Moreover, this model is a part of the reaction model by Kamal [27] that has been used broadly for parameterizing the kinetics of cyanate ester polymerization [28,29].

The experimentally found values of *E_α_* and *A_α_* were used to determine the numerical form of the integral reaction model as follows:

(6) $$g\left(\alpha \right)=\underset{\alpha }{\sum }A_{\alpha }\;J\left[E_{\alpha },T_{i}\left(t_{\alpha }\right)\right]\;\;\;$$

## 4. Results and Discussion

**Figure 3:**
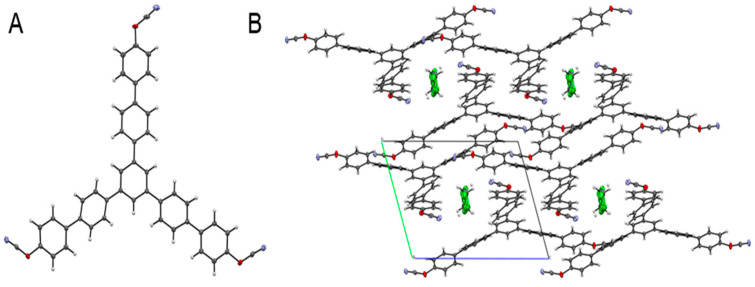
Single-crystal X-ray crystallographic structure of synthesized tricyanate ester **6** (H atoms—light grey, C atoms—dark grey, N atoms—blue, O atoms—red, Cl atoms—green) (**A**). Crystal packing of tricyanate ester **6** view along *a* axis (**B**).

Figure 3A presents the structure of the synthesized monomer as confirmed by X-ray analysis. Crystallization of the target monomer from dichloromethane solution results in the formation of the corresponding solvate containing dichloromethane molecules in the crystalline lattice (Figure 3B).

**Figure 4:**
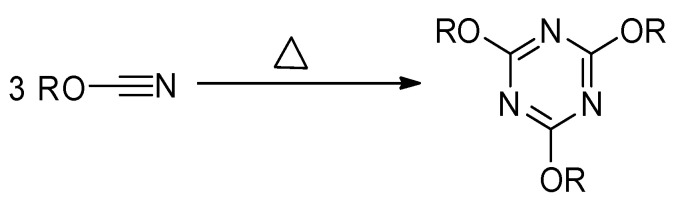
Scheme of cyclotrimerization of cyanate ester.

Thermally stimulated polymerization of the cyanate ester proceeds in the liquid state (i.e., the monomer melt) and results in the formation of highly stable aromatic 1,3,5-triazine fragments as cross-links (Figure 4), which produces significant amount of heat [30]. Thus, the reaction progress is conveniently monitored by DSC.

**Figure 5:**
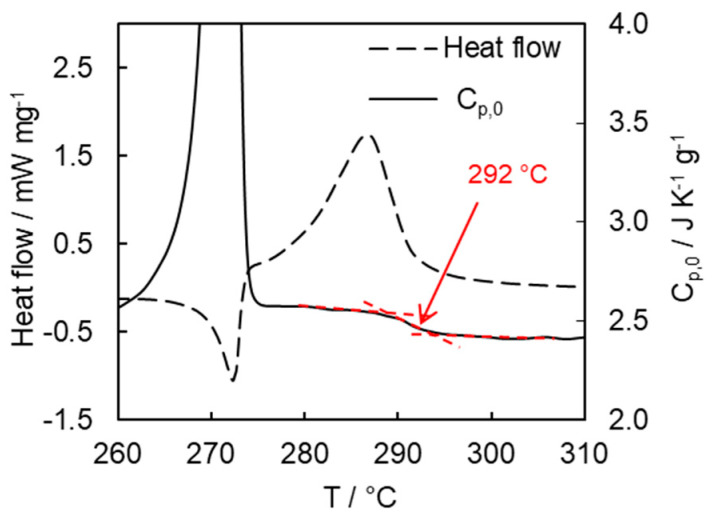
Non-reversing heat flow and quasistatic heat capacity curves for cyanate ester polymerization measured at 1 °C min^−1^ by stochastically modulated DSC (arrow denotes the midpoint temperature of vitrification).

As mentioned earlier, the kinetics of the cross-linking polymerization can be convoluted by a transition from a kinetic- to a diffusion-controlled regime, which is usually observed near the glass transition temperature of the reaction mixture. This transition is accompanied by a change in the kinetic parameters of the polymerization process and usually correlates with vitrification, because the latter is associated with a dramatic increase in viscosity and, thus, with diffusional retardation. To detect vitrification during the cyanate polymerization, temperature-modulated DSC measurements (TMDSC) were employed. The technique separates contributions of the reversing (vitrification) and non-reversing (polymerization) components into the measured total heat flow [31]. Due to the difference in the heat capacities of the liquid and glassy states, vitrification manifests itself as a step change in the temperature dependence of the heat capacity. As shown in Figure 5, the quasistatic heat capacity, *C*_p,0_, determined from the reversing heat flow, reveals such step change. The mid-point of the heat capacity step is found at 292 °C. The exact conversion value related to this transition is difficult to determine because of the partial overlap of the melting and polymerization peaks. However, it is clear that it occurs at the later polymerization stages, probably at α > 0.9. In principle, this means that the respective kinetics of polymerization may undergo a transition to a diffusion-controlled regime at the later polymerization stages.

**Figure 6:**
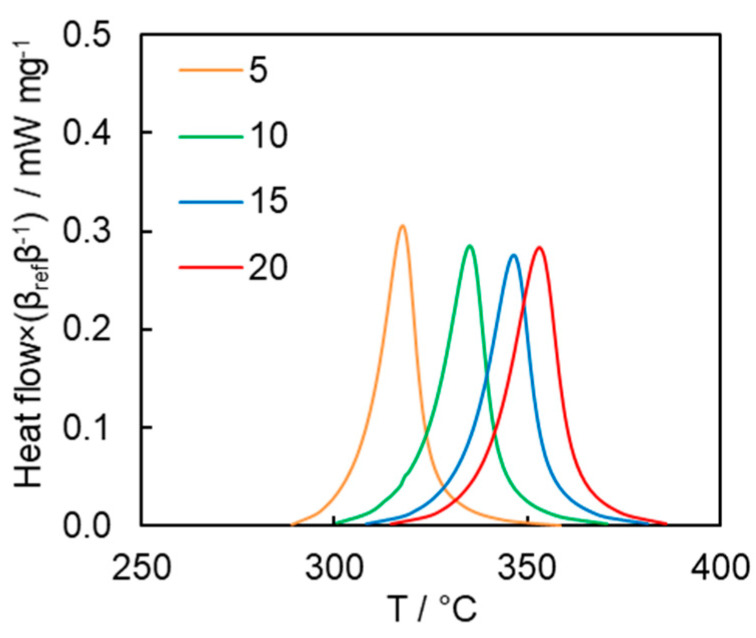
DSC curves for cyanate ester polymerization (numbers denote heating rates in °C min^−1^). The heat flow values at different heating rates were normalized by a factor of 𝛽_𝑟𝑒𝑓_𝛽^−1^, where 𝛽 is the heating rate and 𝛽_𝑟𝑒𝑓_ is 1 °C min^−1^, to facilitate direct comparison of the DSC peaks.

The kinetics of the polymerization process were studied by conventional DSC. Figure 6 presents the DSC curves for polymerization of the cyanate ester at different heating rates. The average heat of polymerization is 282 ± 15 J g^−1^, which corresponds to 185 ± 10 kJ mol^−1^ or 62 kJ mol^−1^ of OCN groups. This is below the reaction heat values of 80–110 kJ mol^−1^ of OCN groups typically reported for mono- and di-cyanate esters [2]. Since the FTIR measurement of the polymerization product does not show the presence of the absorption bands corresponding to unreacted OCN groups, we believe that a possible reason for markedly lower heat values is that the rigid nature of the monomer hinders complete cyclization of the linear reaction intermediates into triazine fragments [32].

**Figure 7:**
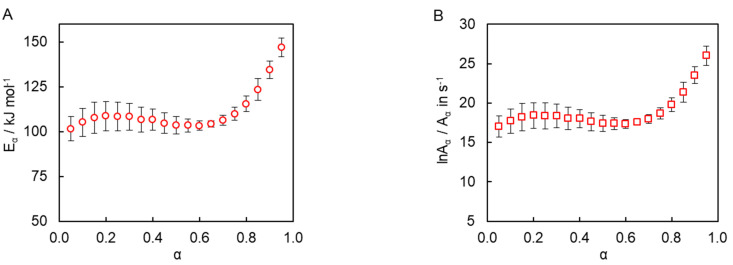
Dependencies of activation energy (**A**) and pre-exponential factor (**B**) as a function of conversion for polymerization of cyanate ester.

Isoconversional kinetic analysis of the measured DSC data was carried out to quantify the reactivity of the synthesized tricyanate ester. The conversion dependencies of the activation energy *E_α_* and pre-exponential factor *A_α_* determined for the polymerization process are presented in Figure 7. A variation of the activation energy with conversion in a range of *α* = 0.05–0.70 is insignificant, i.e., less than 10% of the average *E_α_* (106 ± 6 kJ mol^−1^). The pre-exponential factor also demonstrates a rather small variation (Figure 7B). The average *lnA_α_* value is 18 ± 1. The rise of the activation energy at *α* > 0.70 up to 150 kJ mol^−1^ is likely related to the transition of the polymerization kinetics to a diffusion-controlled regime as was expected from TMDSC measurements. At the same time, the insignificant variation of the kinetic parameters in the conversion range 0.05–0.70 (i.e., in the kinetically controlled regime) indicates that the process appears to be single-step kinetics.

Most commonly, the kinetics of cyanate esters polymerization is treated by a reaction model that combines parallel *n*th order and auto-catalytic reactions (Equation (7)) [6,20,28,29,33,34,35,36,37,38,39].

(7) $$\frac{d\alpha }{dt}=k_{1}\alpha^{m}{\left(1-\alpha \right)}^{n}+k_{2}{\left(1-\alpha \right)}^{n}\;\;\;$$

The constancy of the activation energy in a range of *α* = 0.05–0.70 (Figure 7A) indicates that the polymerization process rate is either controlled by only one reaction step or that the two aforementioned steps possess reasonably close activation energies. Since the cyanate ester polymerization is generally known to involve multiple steps [5], the second case appears more likely. To properly treat such situations, Equation (7) needs to be rearranged by factoring out ${\left(1-\alpha \right)}^{n}$ and $k_{1}$ and replacing $k_{2}/k_{1}$ with B:

(8) $$\frac{d\alpha }{dt}=k_{1}\left(B+\alpha^{m}\right){\left(1-\alpha \right)}^{n}\;\;\;$$

When *E*_1_ ≈ *E*_2_, parameter *B* is a constant equal to the ratio of *A_2_/A*_1_. Then, Equation (8) allows one to identify the reaction model as follows:

(9) $$f\left(\alpha \right)=\left(B+\alpha^{m}\right){\left(1-\alpha \right)}^{n}$$

Equation (8) has an important advantage over the autocatalytic model defined by Equation (5), sometimes referred to as the truncated Sestak–Berggren or expanded Prout–Tompkins model. Practical application of Equation (5) for kinetic simulations requires making an illogical assumption that a reaction starts at some non-zero value of *α* [40]. Unfortunately, the resulting simulations are affected significantly by the choice of the initial non-zero value of *α*. The aforementioned model (Equation (9)) is free of this problem.

Because the constant *B* in Equation (9) is the ratio of pre-exponential factors of two competing reactions, it provides an estimate of how many times the nth-order reaction rate constant exceeds the autocatalytic reaction rate constant. In the case of *B* ≤ 0.1 or *B* ≥ 10, the contribution of one of these reactions to the overall reaction rate constant is less than 10%, which is close to the error of estimating the rate constant. In such situations, the overall process can be considered as a quasi-single-step reaction.

**Figure 8:**
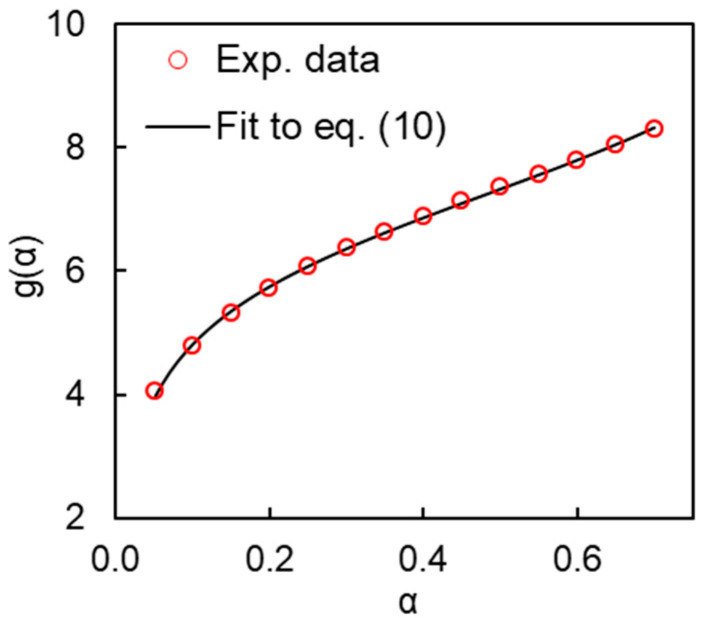
Fitting experimental *g*(*α*) data for polymerization of cyanate ester.

Figure 8 demonstrates the ability of Equation (9) to fit the numerical *g*(*α*) data obtained from the experimental values of *E_α_* and *A_α_* via Equation (6). To perform fitting, Equation (9) is converted to the integral form as follows:

(10) $$g\left(\alpha \right)=\overset{\alpha }{\underset{0}{\int }}\frac{d\alpha }{(B+\alpha^{m}){\left(1-\alpha \right)}^{n}}$$

Clearly, the model provides a good quality fit (Figure 8).

Additionally, the rate data for 0.05 < *α* < 0.70 were fitted directly by Equation (8) to obtain other parameters of the polymerization process. This fitting procedure used *A*_1_, *m*, *n*, and *B* as adjustable parameters. In Equation (8), the temperature dependence of *k*_1_ is introduced via the Arrhenius equation, in which *E*_1_ is kept as a constant equal to 106 ± 6 kJ mol^−1^, i.e., equal to the average *E_α_* value determined by the advanced isoconversional method. Table 1 presents the obtained values of the kinetic parameters. The obtained *lnA*_1_ value is 18.1 ± 0.1, which is practically the same as that determined independently from the compensation effect (18 ± 1). In turn, the value of *B* indicates that *A*_2_ is about 30 times smaller than *A*_1_. The obtained *A*_1_ and *B* values can be used to estimate the value of *lnA*_2_. It is found to be 14.7. Since the *B* parameter is found to be less than 0.1, the contribution of nth order reaction to the overall reaction constant is judged to be insignificant for the reasons explained above. Thus, the reaction can be considered as an autocatalytic quasi-single-step processes.

To account for diffusion that controls kinetics of polymerization at the later stages of the process (*α* > 0.7), we applied an approach that considers the total polymerization rate as the product of the reaction-controlled polymerization rate [*dα/dt*]*_r_* and the conversion-dependend diffusion factor *f_d_*(*α*): [41,42]

(11) $$\frac{d\alpha }{dt}={\left[\frac{d\alpha }{dt}\right]}_{r}f_{d}\left(\alpha \right)$$

**Figure 9:**
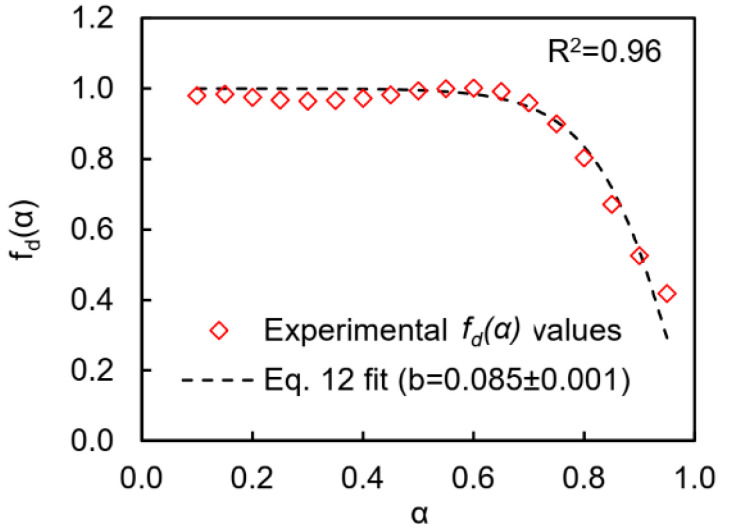
The diffusion factor *f_d_*(*α*) values averaged over all heating rates calculated with kinetic parameters from Table 1 (diamonds) and the best fit by Equation (12) (dashed line).

*f_d_*(*α*) equals unity when polymerization proceeds in a kinetically controlled regime and starts to decrease when it switches to diffusion control. The kinetically controlled polymerization rate can be easily calculated by means of Equation (8) using the kinetic regime parameters (Table 1) and experimental *α* and *T* values. According to Equation (11), the diffusion factor values at the corresponding *α* values are the ratio of the experimental polymerization rate to the calculated polymerization rate in the kinetically controlled regime. As expected, the values of the *f_d_*(*α*) plateau near unity up to conversion of 0.7 then drop to ~0.4 when the polymerization transitions to the diffusion-controlled regime (Figure 9). The Fournier empirical equation [41] was used to describe a dependence of diffusion factor on conversion:

(12) $$f_{d}\left(\alpha \right)=2{\left(1+\exp\left[\frac{\alpha -\alpha_{f}}{b}\right]\right)}^{-1}-1\;\;$$

where *α_f_* is the final conversion and *b* is an empirical parameter. The goodness of the fit of Equation (12) to calculated *f_d_*(*α*) values is shown in Figure 9.

**Figure 10:**
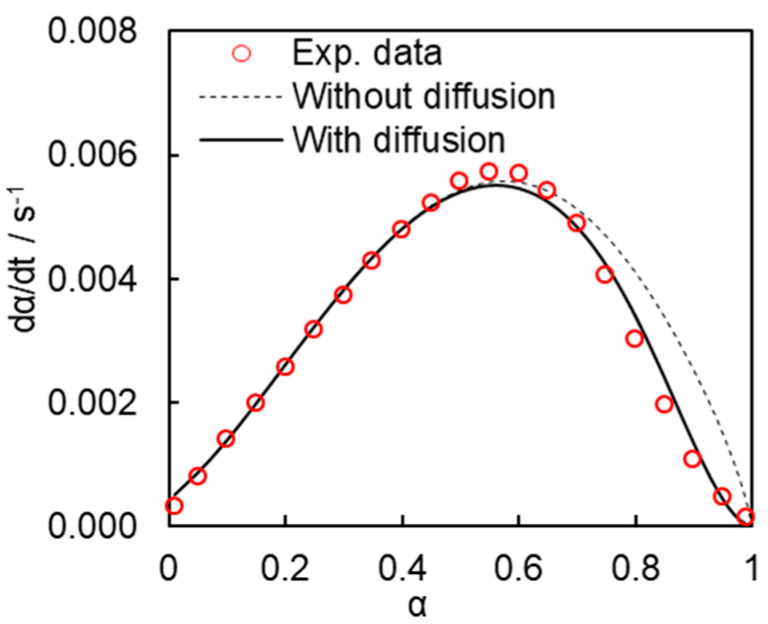
Comparison of the experimental polymerization rate curves (triangles) with those estimated with (solid line) and without (dashed line) accounting for diffusion control.

Figure 10 shows how the rate equations with and without accounting for diffusion (i.e., Equations (8) and (13)) describe the experimental polymerization rate data in the whole range of conversions. As expected, Equation (8) cannot describe the experimental rate outside of the kinetically controlled conversion range, whereas Equation (13) is able to fit the experimental data in the whole range of conversions.

(13) $$\frac{d\alpha }{dt}=k_{1}{\left(1-\alpha \right)}^{n}\left(B+\alpha^{m}\right)\left(2{\left(1+\exp\left[\frac{\alpha -1}{b}\right]\right)}^{-1}-1\;\;\;\right)\;$$

It is instructive to compare the reactivity of the presently synthesized rigid tricyanate ester with other cyanate esters of lower functionality that contan flexible bridging units. The transition of the polymerization from a kinetic- to a diffusion-controlled regime is of particular interest. As previously mentioned, polymerization of the presently studied tricyanate ester transitions to the diffusion-controlled regime at *α* ≈ 0.7. In turn, non-catalyzed polymerization of dicyanate esters with flexible bridging units does not show such transition under non-isothermal conditions, which means that reaction proceeds in a kinetically controlled regime in the whole conversion range [20,30,34].

**Figure 11:**
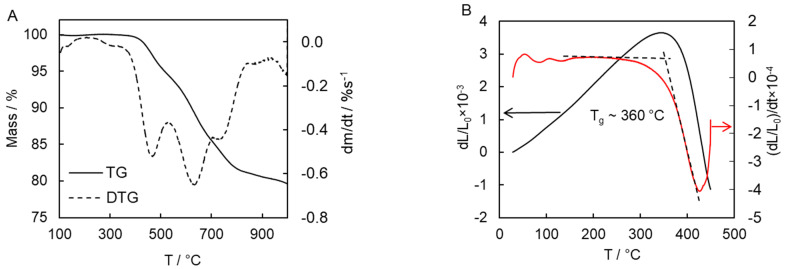
TG and DTG curves of thermal decomposition of polymerization product at 10 °C min^−1^ (**A**); TMA measurements for polymerization product (**B**).

The thermal stability and glass transition temperature of the polymerization product were evaluated by TGA and TMA (Figure 11). Thermal decomposition of cyanate polymers is known to be a complex process. This complexity manifests itself in the multi-step character of the mass loss that reveals the presence of three large peaks (463, 640, and 740 °C) in the derivative TG (DTG) curve (Figure 11A). It should be noted that the thermal behavior of the present polymer product compares favorably with the polymers based on commercially available cyanate esters [43]. For example, the 5% mass loss temperature of the synthesized polymer is 515 °C, which is significantly higher than the 439–457 °C found for polymers derived from commercially available monomers [43]. Furthermore, the char yield at 900 °C for the present polymer is 80%, which is substantially larger than that for the aforementioned polymers (31–63%) [43]. The glass transition temperature of the present polymer is determined as high as 360 °C (Figure 11B), which greatly exceeds the value determined for known crosslinked cyanate polymers (192–289 °C) [3,30,43]. Apparently, such exceptional thermal stability, high glass transition temperature, and char yield are associated with the rigid polyaromatic nature of the cyanate ester monomer [15,16,44,45]. Moreover, one should generally expect that introducing polar groups or groups capable of forming hydrogen bonds into the aromatic rings of the monomer to lead to an increase in *T_g_* of a polymer.

## 5. Conclusions

We synthesized a new rigid tricyanate ester and confirmed its structure by X-ray analysis. Upon heating, the monomer polymerized in the liquid (melted) state. The polymerization process was studied by conventional and temperature-modulated DSC. Temperature-modulated DSC detected vitrification in the later stages of the process, and detailed kinetic analysis revealed corresponding changes in the kinetic parameters of the polymerization process, which were interpreted as a transition from a kinetic- to a diffusion-controlled regime. Moreover, kinetic analysis showed that polymerization in the kinetically controlled regime of the present monomer can be described as a quasi-single-step, auto-catalytic, process. The polymerization product demonstrated exceptional thermal stability, high glass transition temperature, and high char yield. Overall, the obtained experimental results appear consistent with our hypothesis about the effect of the rigidity and functionality of cyanate ester structure on its reactivity and the glass transition temperature.

## Figures and Tables

**Table 1:** Estimated kinetic parameters for polymerization of tricyanate ester **6**.

*E*_1_/kJ mol^−1^	ln(*A*_1_/s^−1^)	*m*	*n*	*B*	ln(*A*_2_/s^−1^)
106 ± 6	18.1 ± 0.1	1.4 ± 0.1	1.2 ± 0.1	0.033 ± 0.01	14.7 *

* denotes the *lnA*_2_ value estimated from the values of *B* and *lnA_1_.*

## Data Availability

The data presented in this study are available on request from the corresponding authors.
